# Seasonality and intensity of airborne Boletus-type spores in relation to land use and weather pattern

**DOI:** 10.1186/s43008-023-00135-4

**Published:** 2023-12-20

**Authors:** Magdalena Wójcik, Idalia Kasprzyk

**Affiliations:** Institue of Biology, College of Natural Sciences, University of Rzeszów, Zelwerowicza 4, 35-601 Rzeszów, Poland

**Keywords:** Aerobiology, Basidiospores, Boletus-type spore, Meteorological parameters, Land use

## Abstract

Forests are a natural source of airborne bolete spores. The timing of sporulation and its intensity as well as the dispersal of airborne spores and in consequence their concentrations depend in particular on the type of land use determining the availability of matter on which they develop and on meteorological factors. The aim of this study was to perform a spatial and temporal analysis of the occurrence of Boletus-type spores in the warm temperate climate of the Northern Hemisphere. An assumption was made that the spore concentrations depend on the type of land cover and weather conditions. The volumetric method was applied to investigate differences in spore concentrations and using spore traps installed at different heights and at locations with different land cover types. Boletus-type spores occurred in the air at high concentrations in late summer and in the autumn. The season start dates and maximum concentrations did not differ significantly between sites and seasons, but the season intensity varied. Higher spore concentrations were usually found in the region with a larger proportion of green areas, including forests. An analysis of the diurnal cycles showed that within 24 h spore concentration reached high levels twice, which was especially noticeable in ground level monitoring. Air temperature and air humidity were the main weather factors affecting the occurrence of airborne spores. This research indicates that when studying the effects of different factors on the concentration of airborne basidiospores, many environmental elements should be analyzed, including the characteristics of habitats in which basidiomycetes grow. Climate, weather, geobotany, and land use type should be taken into account in analysis and interpretation of aeromycological phenomena.

**Supplementary Information:**

The online version contains supplementary material available at 10.1186/s43008-023-00135-4.

## INTRODUCTION

Basidiomycetes produce large amounts of spores, which constitute the important component of the air spora over the world (Elbert et al. 2007). Buller (1909) at the beginning of the twentieth century showed that from 40 million (*Agaricus campestris*) up to 100 million (*Coprinus comatus*) spores are discharged per basidiome per hour. Other authors estimate basidiomycete spore concentrations in 1m^3^ of air from 50 to one million (Levetin 1990; Elbert et al. 2007). Frequently, the spore size does not exceed 10 µm in length (Galante et al. 2011). Spores of many basidiomycetes are ballistospores, that is, spores that are launched into the air as from a catapult (Liu et al. 2017). The average spore launch speed ranges from 0.6 to 1.4 m/s, whereas accelerations are about ten times slower than those characteristic for spore launches driven by turgor pressure, e.g. *Ascomycota* or *Zygomycota* spores (Yafetto et al. 2008; Stolze-Rybczynski et al. 2009). Zoberi (1969) demonstrated experimentally that basidiospores can be launched over a distance from 25 µm to 300 µm, whereas Fischer et al. (2010) report that this distance can be from 9 to 63 times the spore length.

Many species release basidiospores cyclically, while other ones at a certain time of the day or night. Spores released during the day may remain in the air for even several days, whereas those released at night can be deposited within several hours. It should be emphasized that the flight time of the spores depends mainly on turbulence—intense turbulence during the day and weak turbulence at night. The release of spores of some species during the day causes them to be carried to the upper atmosphere, resulting in long flights (Grewling et al. 2022). Spores released at night never reach great heights and fall faster. Because turbulence varies from day to day, the spore release cycle does not coincide with the airborne cycle (Lagomarsino Oneto et al. 2020). Galante et al (2011) prepared a model to describe dispersal indicating the height of the cap, the volume of spores, and the wind speed as the most important factors. In about 95% of the macromycetes tested by them the spores fell within 1 m of the parent basidiomes. Other authors have claimed that spores can be transported in the air for large distances (Gregory 1978; Sadyś et al. 2014), but that depends very much on the spores involved.

Basidiospore production is highly variable, but it is large enough to be recognized as a significant component of the air spora (Hirst 1953). Airborne spores of the following *Agaricomycetes* genera have been found, among others; *Agaricus*, *Agrocybes*, *Bovistella*, *Calvatica*, *Chlorophyllum*, *Clitocybe*, *Coprinus*, *Entoloma*, *Ganoderma*, *Inocybe*, *Pleurotus*, *Russula*, and *Scleroderma* (Kaufert 1935; Levetin 1990, 1991; Oliveira et al. 2010; Quintero et al. 2010; Grinn-Gofroń et al. 2020). Aerobiological studies of basidiospores have focused mainly on *Ganoderma*, which occurs in great numbers and stay airborne for a long time (Almaguer et al. 2014). Information on the occurrence of Boletus-type spores in the air is scarce (Li 2005; Hernández Trejo et al. 2013; Kilic et al. 2020). Boletus-type spore concentrations have been monitored in Spain, Turkey, and the USA (Levetin 1990; Hernández Trejo et al. 2013; Grinn-Gofroń et al. 2020; Kilic et al. 2020), but in addition to research presented by us (Wójcik and Kasprzyk 2023) there are no data regarding the occurrence of fungal spores of this type in the air of the temperate climate zone of the Northern Hemisphere. Existing studies show a decrease in concentrations of airborne spores with a strong wind and an increase in their concentration with increasing air temperature. The nature of both these relationships and the relationships with other meteorological elements require detailed analysis (Foti et al. 2008; Castillo et al. 2013; Rivera-Mariani et al. 2014).

The content of fungal spores in the air largely depends on the availability of biomass on which fungi can develop, which, according to some authors, is more important than climatic conditions or weather patterns (Skjøth et al. 2016). It has been proven that agricultural land and its management are crucial for the occurrence of conidia of *Alternaria*, known cereal pathogens (Skjøth et al. 2016; Olsen et al. 2019). *Basidiomycota* occur in forests, meadows, woody and shrub veretation areas, natural vegetation areas and even agricultural areas. All these areas are potential sources of airborne fungal spores. Sadyś et al. (2014) and Grinn-Gofroń et al. (2021) confirmed that the concentrations of *Garnoderma* basidiospores in the air depend on the area covered by forests, and on tree density. Boletes are ectomycorrhizal fungi occurring in deciduous, coniferous and mixed forests (Wang et al. 1995, 1998). Grinn-Gofroń et al. (2020) indicated that the main source of *Boletus* spores in the air of Turkish Black Sea region is coniferous forests. However, still little is known about the origin and proportion of airborne spores, hence the need to undertake this research was justified.

A topic poorly explored is the change in fungal spore concentrations in the air with altitude. There is a known tendency to decrease the concentration of air spora components, but the intensity of this phenomenon depends on particle size, dispersion ability, and the proximity of spore sources (Khattab and Levetin 2008; Damialis et al. 2017). However, as Dąbrowska-Zapart and Niedźwiedź (2020) point out, some fungal spores, e.g. basidiospores of *Ganoderma*, show the opposite pattern, therefore, one of the challenges of our research was to investigate this topic for Boletus-type spores.

**Fig. 1:**
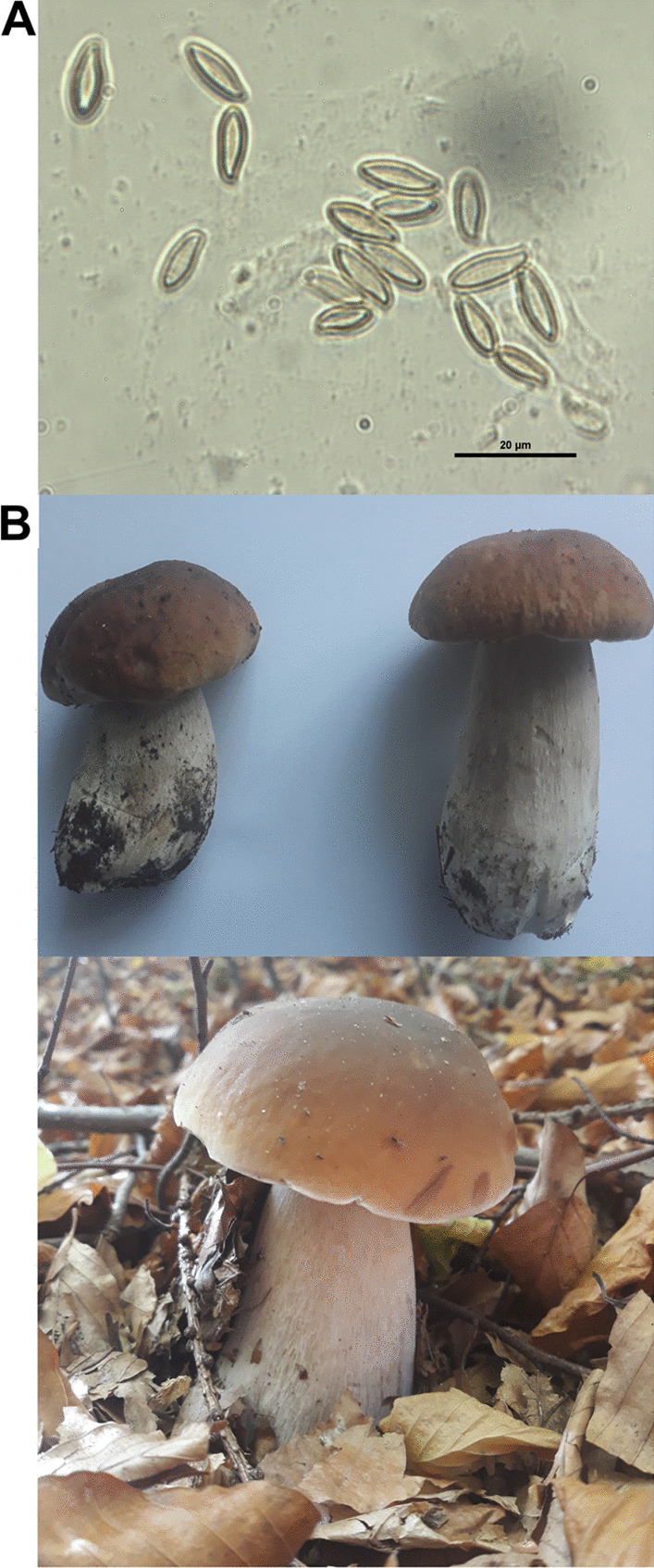
Spores (**A**) and basidiome (**B**) of *Boletus edulis* collected in the study area

The literature review presented above indicates that bolete aerobiology is very poorly understood. The aim of this study was to perform a spatial and temporal analysis of the occurrence of airborne Boletus-type spores in the warm temperate climate. The implementation of this aim allowed us to test the following hypotheses: (1) the seasonal and diurnal cycles of airborne spores do not change spatially; (2) the concentration decreases with the altitude at which the measurement is carried out; (3) the type of land cover is an important factor influencing intensity of the seasons, and the main sources of airborne Boletus-type spores are woodlands; and (4) the spore content in the air is dependent on the weather, especially temperature and rainfall (Fig. 1).

## MATERIAL AND METHODS

### Study area

#### Location

**Fig. 2:**
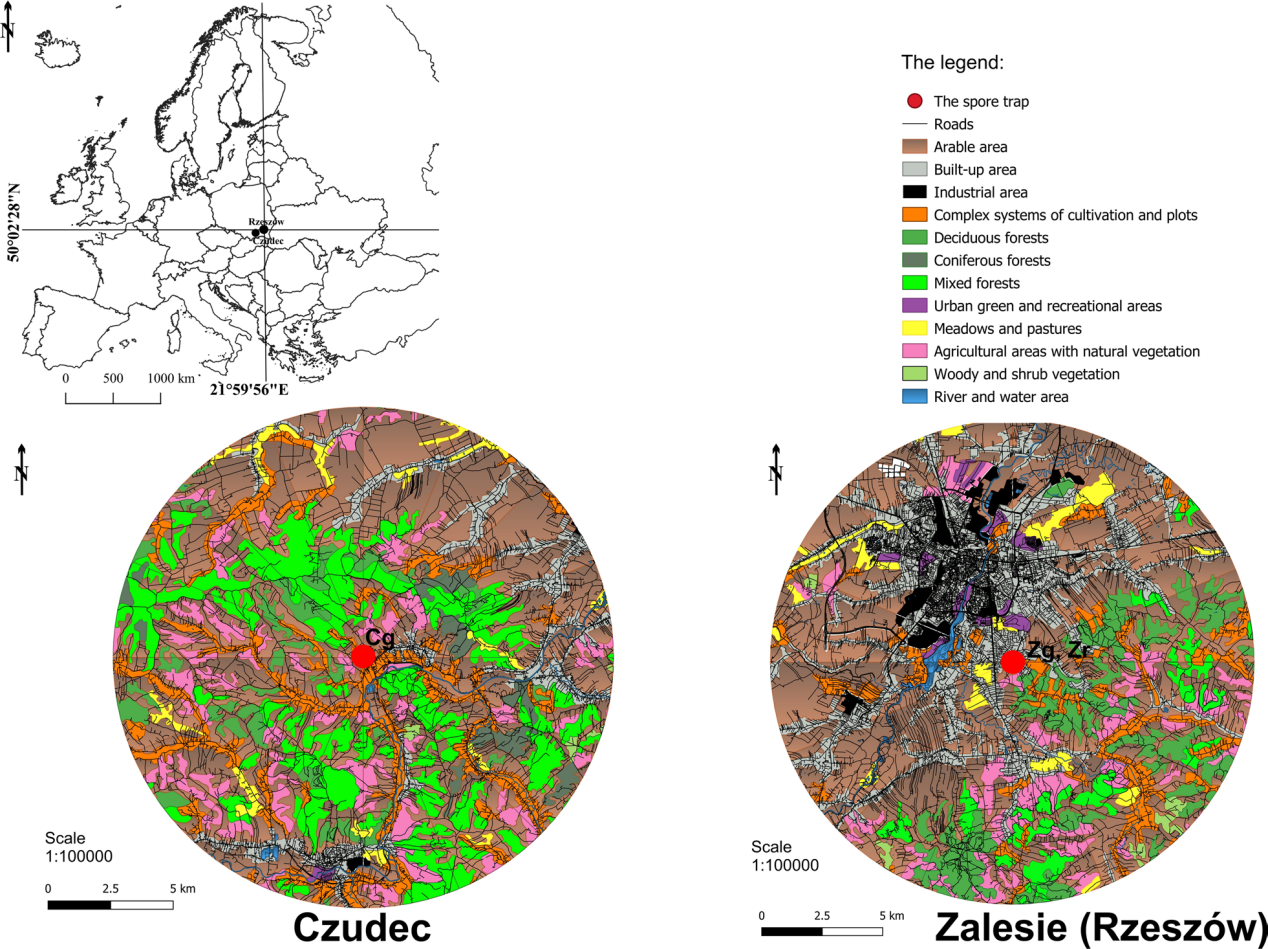
Location of the study area

An aerobiological study was conducted in south-eastern Poland in the city of Rzeszów ( Zalesie district), and in the village of Czudec about 20 km south-west from Rzeszów (Fig. 2). Both study sites are located in the Strzyżów foothills (Ćwik et al. 2021) and differ in area and land use (Additional file 3: Table S1).

Rzeszów is the region’s largest city. Vegetation covers slightly over 40% of the city’s area. The urban greenery consists mainly of lawns, 10 parks, playgrounds, and sports fields. The Lisia Góra Nature Reserve, in which there are centuries-old oak stands, is the only urban forest. In Czudec, arable land is interwoven with tree and shrub communities and wasteland, with natural vegetation predominant. Anthropogenic areas are dispersed, but concentrated along the road network.

Within a 10 km wide buffer from Rzeszów and Czudec, vegetation covers 73.2% and 92.3% of the area, respectively, of which agricultural areas constitute 40 and 42%, respectively. The percentage of forests and woodland, that favor the occurrence of *Boletaceae*, in Czudec is twice that in the Rzeszów buffers, accounting for nearly 27% of the area. The forests of the Rzeszów region occur mainly from the NE to the SSW sectors, while in the Czudec region they are distributed in all directions (Additional file 3: Table S1).

#### Climate

**Fig. 3:**
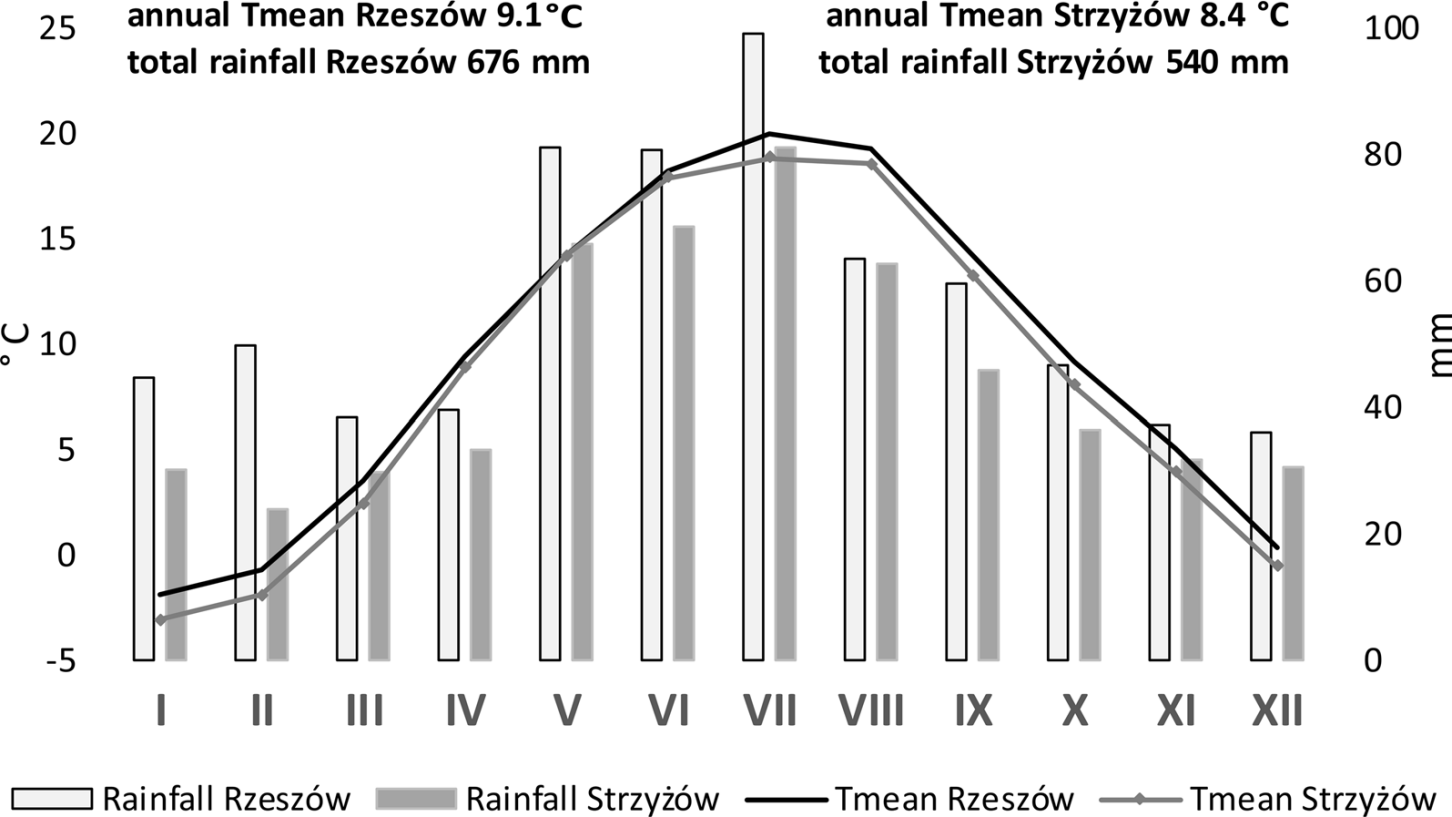
Climatic diagrams for the two research areas

This region is situated in a transitional warm temperate climate. A detailed description of the climate of the region in which the study sites were located was made based on an analysis of meteorological data for the period 2002–2021 from two cities: Rzeszów and Strzyżów, located at a distance of about 10 km from Czudec. Over this period, the mean air temperature and annual sum of rainfall in Rzeszów were higher than in Strzyżów. In both regions the highest temperature was recorded in July, the temperature in Rzeszów being higher by 1.08 °C than in Strzyżów. The lowest mean temperature in Rzeszów and Strzyżów was recorded in January and it was − 1.92 °C and − 3.1 °C, respectively. In each month, the amount of rainfall in Rzeszów was higher than in Strzyżów. The highest rainfall occurred in July (Fig. 3).

### Aerobiological monitoring and data analysis

#### Aerobiological methods

The Boletus-type spore content (Fig. 1) in the air was measured from the beginning of March to the end of November in the period 2019–2021 at both urban locations and in the one rural one. For air monitoring, the volumetric method was applied using a Hirst-type sampler. Two samplers were located at a height of 1.5 m above ground level, respectively in the city of Rzeszów, the Zalesie (Zg) district, and in the village of Czudec (Cg). At the Zalesie site, right next to site Zg, a trap was additionally installed on the roof of a building (Zr) 12 m above ground level. The volumetric sampler sucks 10 l of air per minute. Inside the sampler, there is a drum with Melinex tape wound on it and the drum rotates 2 mm per hour using a clockwork mechanism. Fungal spores, sucked into the sampler together with air through a 2 mm wide orifice, stick to the tape which is smeared with a sticky silicone. The tape was replaced once a week at a fixed hour.

In the laboratory, the tape was cut into 48 mm long pieces, corresponding to individual days, and microscope slides mounted in glycerol gelatine stained with fuchsin were prepared. Spores were identified and counted along one longitudinal transect divided into 24 2 × 2 mm fields corresponding to subsequent hours using a light microscope with a magnification of 600 × . Taking into account the magnification used and the surface area of the slide analyzed, the average daily concentration of Boletus-type spores per 1 m^3^ of air was calculated according to the formula presented by Galán et al. (2017). According to the current classification, Boletus-type spores are mainly represented by fungal spores of the genera *Boletus*, *Chroogomphus*, *Gomphidius*, *Leccinum*, *Suillus*, and *Xerocomus* (Hernández Trejo et al. 2013) and hence the term Boletus-type is used in this article.

#### Data processing and statistical methods

The first stage of the data analysis was to fill data gaps. For this purpose, the lineal method was applied. Basic descriptive statistics were calculated for the database so prepared. The airborne spore season was determined by the 90% method, which is considered to be the best one for this type of palynomorph by Olsen et al. (2019) and Grinn-Gofroń et al. (2020). The start date is the day on which the total annual spore count exceeds 5%, while the season end date is the day when the total annual spore count exceeds 95%. The season start (st.dt) and end dates (en.dt) as well as the season length (ln.ps) were determined. Next, percentage of days in the season with at least one spore (fr), maximum concentration (pk.val) and the day of the peak value (pk.dt) seasonal spore integral (SSIn − total sum of spores in the season), and the total sum of spores in the entire period of the study (smtt) were calculated. Additionally the number of days with more than 100 spores (daysth) were calculated. All calculations were done using the AeRobiology package. The coefficient of variation (CV%) was calculated to describe the variation of these parameters. In order to check the synchronization of the spore season patterns between the sites, Spearman’s rank correlation was performed.

### The fungal spore concentrations versus land type cover

#### Land cover analysis

Land cover maps were prepared using QGIS software version 3.4.12 based on Corine Land Cover 2018, in which 12 land cover types were marked: mixed, coniferous and deciduous forests, woody and shrub vegetation, meadows and pastures, agricultural areas with natural vegetation, arable area, water, complex systems of cultivation and plots, built-up area, urban green and recreation area, and industrial area. The study sites were designated and subsequently the area of the individual land cover types was calculated within a 10 km buffer, which was considered to be sufficient due to the fact that basidiomycete spores are dispersed over small distances (Zoberi 1969; Fischer et al. 2010) and the installation of two stations at a low height.

#### Data analysis

The impact of the type of habitat on spore concentrations was examined using the Spearman's rank correlation, adopting the approach proposed by Grinn-Gofroń et al. (2020). The buffers around the study sites were divided into 16 smaller sectors, corresponding to the directions of the wind. For these sectors, the area occupied by each of above described types of land cover (in ha) was calculated (Additional file 3: Table S1), what was the independent variable in further correlation analysis. The number of spores on days with each of the 16 wind directions was then summed. This made it possible to calculate the correlation coefficients between the sum of airborne spores and the area occupied by a specific type of land use in each of 16 sectors for each site and year separately. This allowed us to indicate from which type of land cover the spores were transported in high concentrations. Then, using Principal Component Analysis (PCA), we checked when (years) and where (sites) the above-described relationships were the strongest. It was necessary to reduce the large number of land cover types by combining some, hence 4 types were distinguished, i.e. forests (F) (mixed, coniferous and deciduous forests, woody and shrub vegetation), cultivated areas (C) (arable area, complex systems of cultivation and plots), areas of green vegetation (V) (meadows and pastures, agricultural areas with natural vegetation), and anthropogenic areas (A) (built-up, urban green, recreation area, and industrial areas). The assumption was made that the areas designated with the symbols F and V were favorable for the occurrence of boletes.

### Circadian periodicity

For the analysis of the diurnal cycle, days were selected according to the following criteria: no rainfall, where the number of recorded spores for one day (raw data) exceeded 50, and where the number of spores exceeded 10 in at least one hour. The hour with the maximum number of spores was determined for each day, but if the numbers at different hours exceeded 90% of this maximum value, they were considered to be additional peaks.

Based on the data prepared in this way, circular statistics methods were applied, calculating the angular mean hour in which the maximum concentrations occurred most frequently with a 95% confidence interval. Such an analysis was made for each site separately. To confirm the significance of the calculations, statistical tests were performed to prove that the empirical data fitted the models built (Watson's U2 test, Rayleigh's R test).

The sum-of-sinusoids function with the option of determining the period was used to describe the daily cycles. The cycles can be defined using cosine, sinus or free adjusted models. The best model had the lowest value of AIC (Akaike Information Criterion) estimator, which inform about the models’ information loss. This procedure was performed using relative values (percentages).

### Fungal spore concentrations versus meteorological parameters

In the city, immediately next to the air monitoring samplers (Zg, Zr), an automated weather station was located making measurements of minimum, maximum, and mean temperatures (Tmin, Tmax, Tmean), relative air humidity (H%), wind speed (WS [m/s]), wind direction (WD), pressure (P), rainfall (R), and solar radiation (SR) (Additional file 1: Fig. S1, Climate data: Poland). For the site in the countryside (Cg), meteorological data for the period 2020–2021 were obtained from an automated weather station installed immediately by the volumetric sampler, which measured the same parameters as that in Rzeszów. For the year 2019, data recorded by a weather station of the Institute of Meteorology and Water Management located in Strzyżów, 10 km away from site Cg, were used (Additional file 2: Fig. S2). The minimum, maximum, and mean temperatures (Tmin, Tmax, Tmean), and rainfall (R) (https://www.imgw.pl/) were used for the analysis.

The meteorological parameters and aerobiological data did not represent a normal distribution and therefore non-parametric statistical tests were employed in the data analysis. To calculate the effects of the weather factors on daily spore concentrations, Spearman’s rank correlation was applied. To this end, the spore concentrations were analyzed in relation to the weather factors on a given day and on two preceding days.

For all statistical tests, the level of significance α ≤ 0.05 was accepted. For circular statistics, PAST ver. 4.03 software was used, while other statistical tests were performed using Statistica 13.

## RESULTS

### Yearly variations of the seasonality and intensity of airborne Boletus-type spores

The pattern of seasons and their intensity were characterized by temporal variability. The greatest differences related to intensity. The season timing and the peak value date were characterized by distinctly smaller variation (Table 1).

**Table 1:** Characteristics of Boletus type spores season in Cg—Czudec ground, Zg—Zalesie (Rzeszów) ground, Zr—Zalesie (Rzeszów) roof in 2019–2021

Seasons	st.dt	st.jd	en.dt	en.jd	fr	ln.s	sm.tt	SSIn	pk.val	pk.dt	pk.jd	daysth
Cg												
2019	21 VI	172	30 IX	273	63.6	102	15,442	13,986	1949	25 VIII	237	28
2020	25 VII	206	06 XI	310	45.8	105	3183	2877	170	18 IX	261	5
2021	28 VII	209	18 IX	261	40.4	53	8765	8004	748	15 IX	258	22
Zg												
2019	13 VIII	225	19 X	292	46.5	68	6395	5767	577	29 IX	272	16
2020	06 VII	187	06 XI	310	44.7	124	2727	2480	209	31 VIII	243	3
2021	14 VII	195	28 IX	271	42.2	77	3964	3572	267	10 IX	253	12
Zr												
2019	30 VI	181	10 X	283	53.5	103	7488	6771	484	29 VIII	241	21
2020	01 VII	182	31 X	304	50.9	123	3567	3247	239	30 VIII	242	9
2021	15 VII	196	25 IX	268	42.9	73	5151	4684	420	10 IX	253	15

*Seasons* year of the beginning of the season, *st.dt* start of the season (date), *st.jd* day of the year of start season, *en.dt* end of the season (date), *en.jd* day of the year of end season, *fr* frequency (%), *ln.s* length of the season (days), *sm.tt* total sum, *SSIn* sesonal spores integral, *pk.val* peak value, *pk.dt* day of the peak value (date), *pk.jd* day of the year of peak value, *daysth* number of days with more than 100 spores

**Fig. 4:**
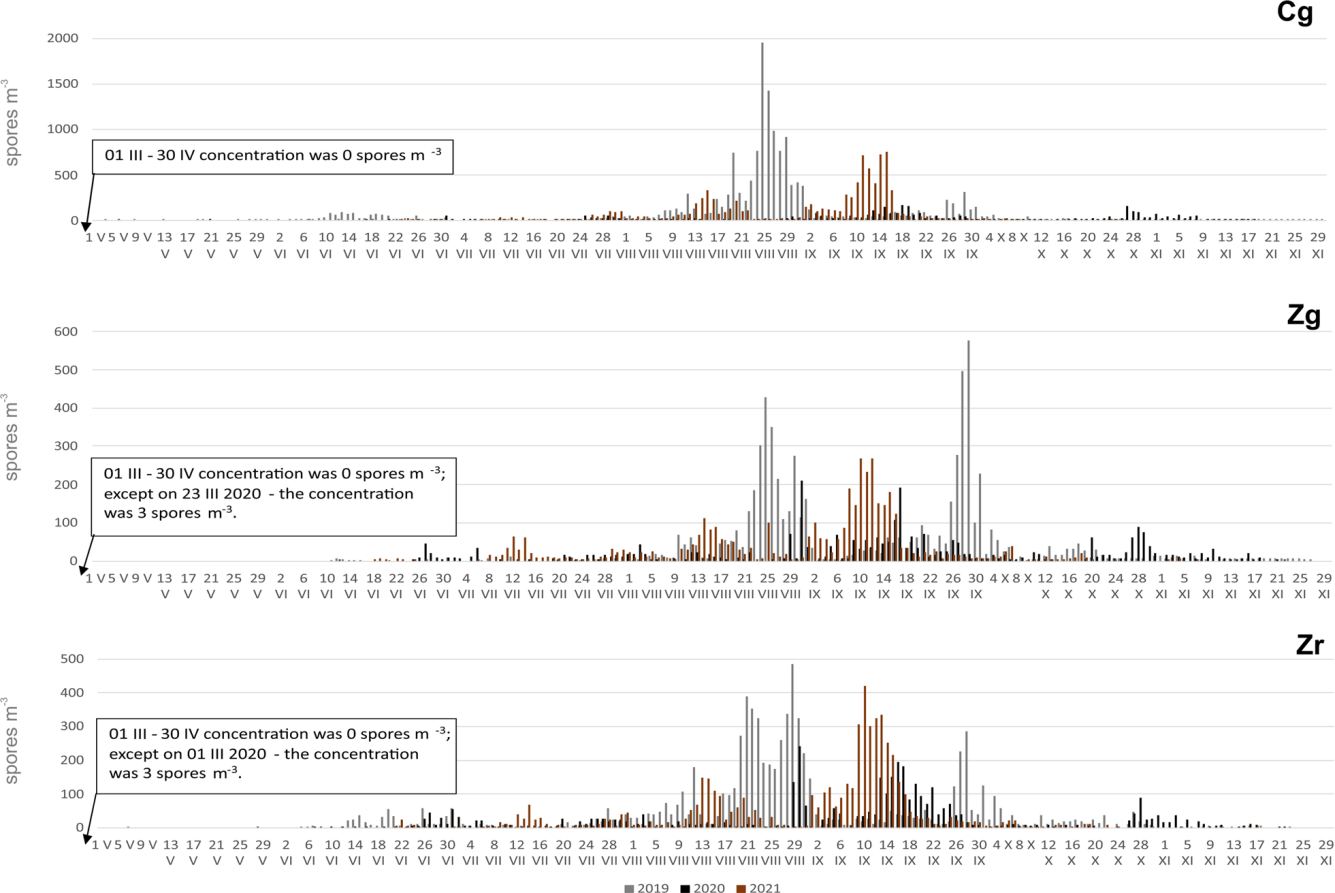
The patterns of the daily concentrations of Boletus-type spores in the air of sampling sites in 2019–2021

**Fig. 5:**
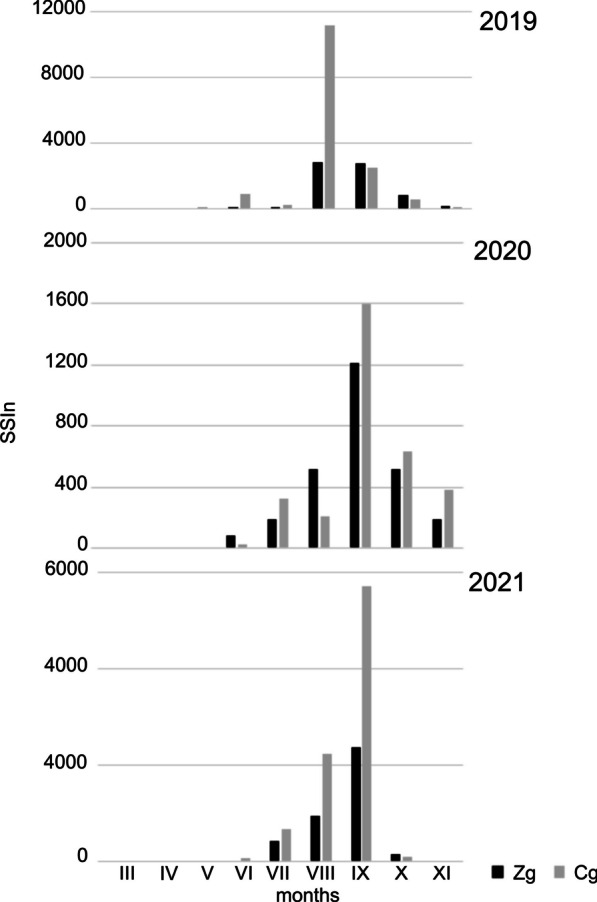
Seasonal spore sum (SSIn) values in the subsequent months in 2019–2021 at two sampling sites located at ground level (Cg, Zg)

**Fig. 6:**
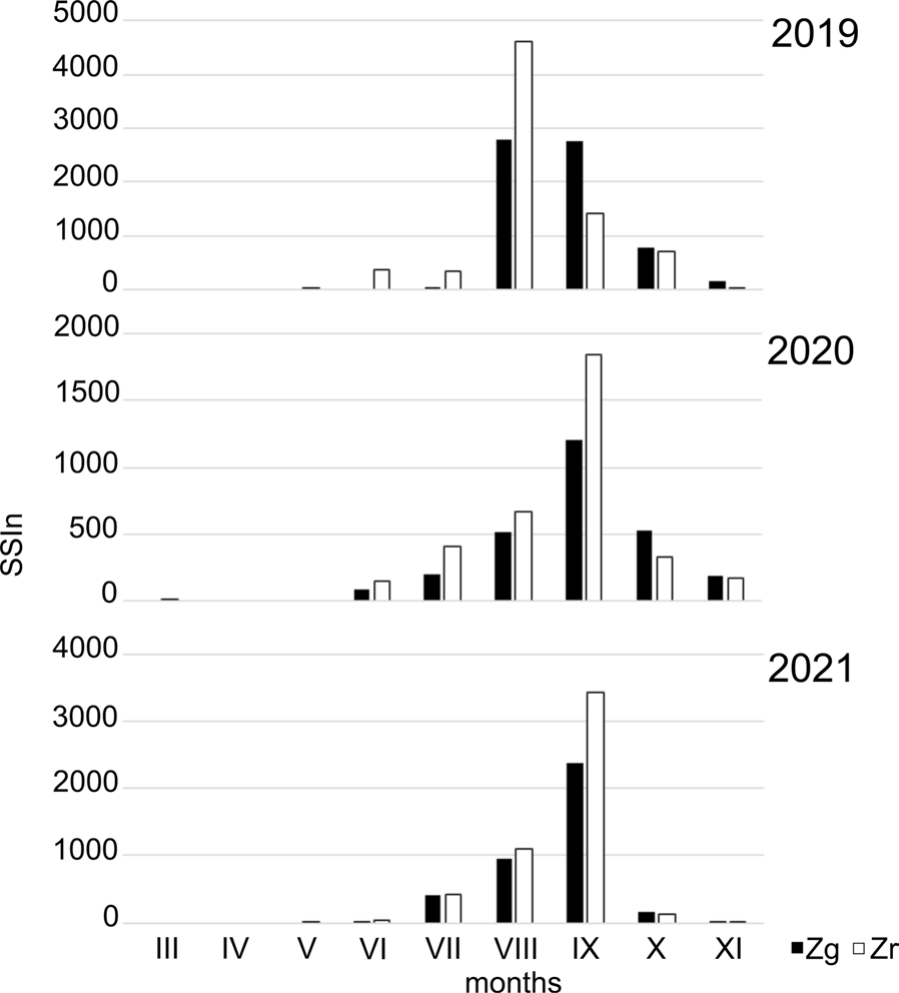
Seasonal spore sum(SSIn) values in the subsequent months in 2019–2021 at two sampling sites located in Zalesie at ground level (Zg) and roof level (Zr)

At each site, the number of spores recorded during the season was highly variable. CV values (coefficient of variation) ranged from 36.2% for Zr to 67.0% for Cg, where in 2019 this value was 13 986, while in 2020 it was almost five times lower. In other locations, SSIn values for 2020 differed in a similar way, although not on such a scale—only two times lower. The maximum concentration values were even more diverse. Extreme results were obtained for Cg, where in the last year of our research this value was almost 100 times lower than in 2019. Despite such large differences in concentrations, the variability of the dates of seasons was much smaller, for Zr the differences between years were only 12 days. The dates of the seasons, especially their endings, were characterized by little variability (Table 1). With respect to the seasonality of the occurrence of spores in the air at a given site, the course of season differed each year. At site Cg, spore seasons were characterized by two or three periods of marked increases in concentrations. In the Zg area, the 2019 season was characterized by two periods of high concentrations: in the second half of August and at the end of September. The following year, the entire season was irregular and concentrations were the lowest. Even though 2021 saw the lowest frequency, this season was more condensed. In mid-September, a significant increase in concentrations was recorded. In Zr, the highest concentrations were observed in August (2019) or September (2020 and 2021). During the period covered by our research, the course of the seasons was most similar (Table 1; Figs. 4, 5 and 6).

In summary, for all sites 2020 was the year with the lowest spore concentrations; the seasons ended latest, were irregular, and the longest. The concentrations rarely exceeded 100 spores per cubic meter. In 2019, the concentrations were highest and while 2021 was characterized by the most compact season.

### Spatial analysis

The synchronization of airborne spore seasons between the study sites was strong, as illustrated by the high values of Spearman’s correlation coefficient (r_s_). The patterns of the seasons were the most similar in 2021 and the correlations between all the sites were greater than 0.8. In the other years, the season patterns were also similar and the correlation coefficient values were never lower than 0.6 (Table 2).

**Table 2:** Synchronization of the occurence of the Boletus type spores in the atmosphere of three sites (Cg, Zg, Zr) in 2019–2021

Sites	r_s_
2019	
Cg/Zg	0.739
Cg/Zr	0.825
Zr/Zg	0.702
2020	
Cg/Zg	0.792
Cg/Zr	0.762
Zr/Zg	0.790
2021	
Cg/Zg	0.858
Cg/Zr	0.862
Zr/Zg	0.864

The differences between the sites with the samplers located *at ground level* in the two regions with different land use relate to above all intensity. In 2019, from May to August, the spore concentration in the countryside was higher than in the city, whereas from September to October the situation was opposite (Fig. 5). The SSIn values confirm differences in season intensity and at site Cg the value of this indicator was more than twice that at site Zg (Table 1). In 2020 the season timing and the frequency of occurrence were similar. The spore concentration was higher in the countryside (Cg) in July and from September to November. In 2021 the spore concentration at station Cg increased from May to September and was higher from the concentrations recorded during these months at site Zg (Fig. 5). The highest spore concentration was observed in the first half of September at both locations, but the peak concentration found in the countryside was almost three times higher (Table 1). The SSIn values clearly differed since at site Cg this value was twice higher than at site Zg (Table 1). The differences in season dates were slightly smaller. The differences in dates were most noticeable in 2019, when at the Cg site the airborne spore season began in June, almost two months earlier than at the Zg site. In other years the differences were about 2 weeks. The dates of the end of the season are slightly less volatile, in 2020 it was the same date.

The spore concentrations at the stations located in the city *at different heights* (Zr and Zg) also varied. Both the SSIn values and the number of days with a concentration above 100 spores/m^−3^ were always higher for Zr. In 2019 spores were recorded on the roof much earlier than at ground level and high concentrations were observed in August (Fig. 6). In 2020 both locations showed similarity in season timing and in the frequency of airborne spores (Table 1). The days on which the highest concentrations of airborne Boletus-type spores were found at both stations were in the last days of August. In 2021, at both sites the season started with a one-day difference (July 11–July 12) and the season end dates were also similar—the end of September. The highest spore concentrations occurred on the same date—September 10 (Table 1). From May to September, the spore concentration on the building roof was higher than at ground level. In October and November, on the other hand, higher spore concentrations were noted at site Zg (Fig. 6). No differences were found in the frequency of airborne spores which were 42.2 (Zg) and 42.9% (Zr), respectively.

### Spore concentrations relative to land cover types

**Fig. 7:**
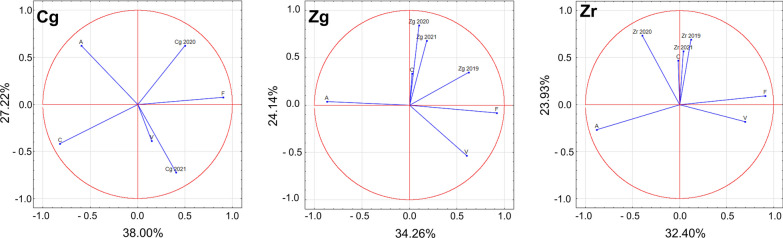
The relationships among airborne spores concentrations in 2019–2021 and four land cover types at three sampling sites (V—areas of green vegetation, F—forests, A—anthropogenic areas, C—agriculturally used areas)

A detailed analysis of land cover allowed potential sources of fungal spores to be identified (Additional file 3: Table S1; Figs. 2, 7). Within a 10 km buffer from station Zg, only 13% of the area was covered by habitats theoretically favoring the occurrence of boletes. The correlation between the area occupied by a specific type of vegetation in the buffer sectors designated according to wind directions and the number of spores was found to be significant only in the case of woody and shrub vegetation. The value r = 0.519 proves that this type of vegetation could have been a source of spores found in the air. Near Czudec, the area favoring the occurrence of boletes was much larger because it accounted for 27% of the 10 km buffer. In 2020 the number of spores was shown to increase when the wind was blowing from the directions where the area was covered by deciduous forests (r = 0.550) as well as by complex systems of cultivation and plots (r = 0.510). The relationship between the number of spores and the arable area was negative (r = −0.519).

In order to obtain a comprehensive view on the nature of the investigated phenomenon, a multidimensional analysis (PCA) was performed. This technique indicated that at site Cg, in 2020 Boletus-type spores originated from forests (F), while on the other hand, their presence could not be linked to agriculturally used areas (C). In 2021, in turn, areas of green vegetation (V) were the main source of spores, whereas anthropogenically transformed areas (A) to the least extent. At site Zg, areas of green vegetation (V), including crops, were the source of spores in each year. The analysis reveals that sources of spores should not be sought in anthropogenic areas. The presence of spores at site Zr in the years 2019 and 2021 was related to agricultural areas (C) and more weakly to forests (F), but it was not associated with anthropogenic areas (A) (Fig. 7).

### Circadian periodicity

**Fig. 8:**
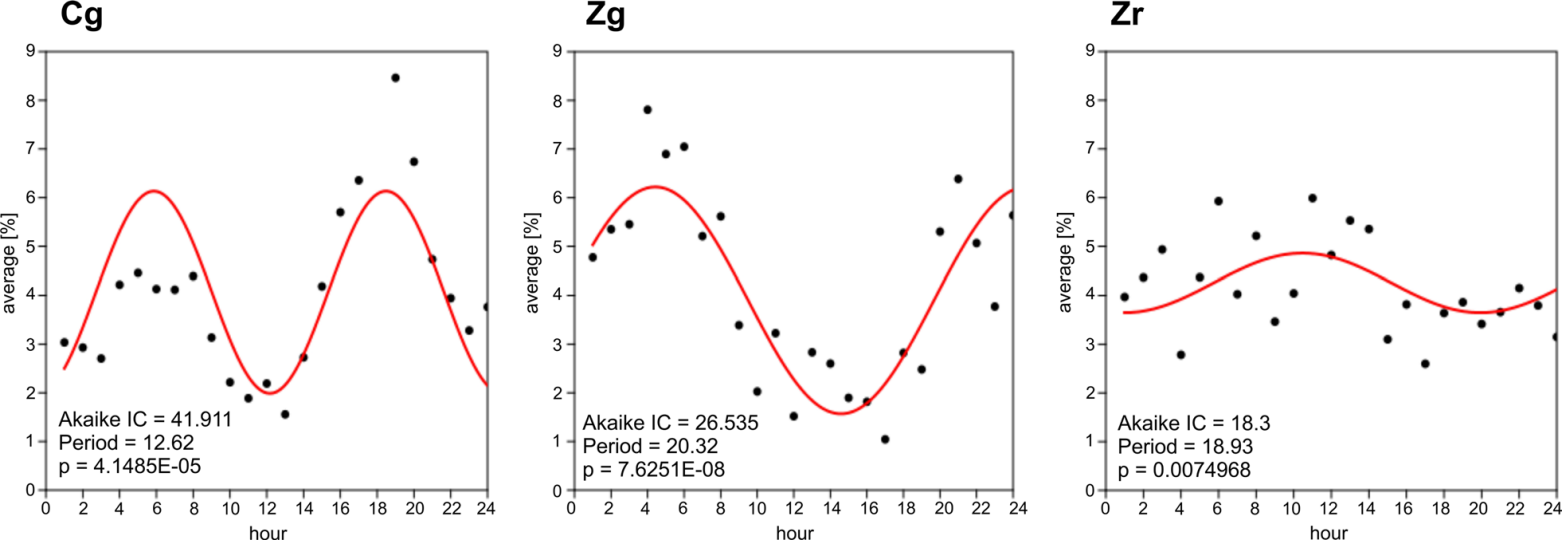
Diurnal periodicity of airborne Boletus-type spores

The analysis of the diurnal cycles performed for all sites showed the existence of two periods within 24 h during which Boletus-type spores occurred in high or relatively high concentrations in the air. The first one was observed at early morning hours, while the other one in the evening or at night (Fig. 8).

At site Cg, two periods with maximum airborne occurred at about 13-h intervals. The first peak occurred at about 6 am, from 7 am the airborne spore concentration clearly decreased, while at 12 am very low concentrations were found. In the afternoon, the airborne spore concentration increased again, at 7 pm reaching its peak value. At site Zg, the first increase in the concentration was observed at about 4 am and subsequently the spore concentration decreased until 2 pm. A second distinct peak occurred at midnight. At site Zr, periods of elevated concentrations recurred every about 18 h. The first peak of the spore concentration was recorded at 10 am, thus later than at the stations located at ground level. In the afternoon, the concentrations dropped, while at night a slight increase in concentrations was observed. Unlike the cycles observed at ground level (Zg), the differences in concentrations between the highest and lowest values were not large (Fig. 8).

The results of circular statistics demonstrate that the maximum Boletus-type spore concentrations at the individual study sites occurred at different hours. At site Cg, the highest concentrations were recorded at night, at site Zr during the day and at night, but at both sites, on average, at early night, at 10 pm and 8 pm, respectively. In the case of site Zg, high spore concentrations were observed after midnight and early morning. The highest spore concentrations were most often observed at 3 am (Table 3).

**Table 3:** Circular statistics results for the frequency of peak values within 24 h at the three study sites (NS—not statistically significant)

Station	Mean hour	95% confidence	Kappa	Watson's U2 test	p	Rayleigh's R test	p
Cg	10 pm	9–12 pm	1.012	1.307	< 0.005	0.451	0.0003
Zg	3 am	2–5 am	1.593	0.029	> 0.5	0.636	0.0014
Zr	8 pm	2–12 pm	0.187	0.072	0.01 < p < 0.025	0.093	0.8373

### Effect of meteorological factors on spore concentration

Boletus-type spore concentrations were correlated to meteorological factors. At all the sites, mean, minimum, and maximum temperature was positively correlated to real-time spore concentrations. Increased airborne spore concentrations were correlated to thermal conditions prevailing on the days preceding the day of measurement. At site Cg, an increase in wind speed in real time and one or two days before the day of measurement promoted a fall in the airborne spore count. Other meteorological factors were not found to affect statistically significantly airborne spore concentrations at this site. At site Zg, humidity and rainfall had an impact on the phenomenon studied. The first parameter favored an increase in spore concentrations in real time and one or two days before the occurrence of the concentration in all three study years, whereas rainfall was negatively correlated to concentrations in 2019 and 2020. Wind speed was negatively correlated to spore concentrations in real time and one or two days before the occurrence of the concentration. The effect of humidity and wind speed was the same at site Zr. Rainfall was associated with a reduction in concentrations, but only in 2019 (Table 4).

**Table 4:** Spearman correlation coefficients expressed the strenght of the relationships between daily concentrations and daily meteorological parameters in actual day (n) and in the previous days (n − 1, n − 2) in 2019–2021

	2019			2020			2021		
	N	n − 1	n − 2	n	n − 1	n − 2	n	n − 1	n − 2
*The parameter*									
Station	Cg								
Tmean (°C)	NS	NS	NS	− 0.210	NS	− 0.194	NS	NS	NS
Tmax (°C)	NS	NS	NS	− 0.199	NS	NS	NS	NS	NS
Tmin (°C)	NS	NS	NS	− 0.218	− 0.19714	− 0.206	NS	NS	NS
Humidity (%)	–	–	–	NS	NS	NS	− 0.378	− 0.335	NS
Dew point (°C)	–	–	–	− 0.266	NS	NS	NS	− 0.274	NS
Wind speed (m/s)	–	–	–	NS	NS	NS	− 0.345	NS	NS
Preasure (hPa)	–	–	–	0.378	0.381	0.367	0.518	0.560	0.506
Rainfall (mm)	NS	NS	NS	NS	− 0.203	NS	− 0.297	NS	NS
Station	Zg								
Tmean (°C)	NS	0.331	NS	NS	NS	NS	NS	NS	NS
Tmax (°C)	NS	0.521	NS	NS	NS	NS	NS	NS	NS
Tmin (°C)	NS	NS	NS	NS	− 0.223	− 0.265	− 0.410	− 0.356	− 0.259
Humidity (%)	NS	NS	NS	NS	− 0.200	NS	NS	NS	NS
Wind speed (m/s)	NS	NS	NS	NS	NS	NS	− 0.359	− 0.275	NS
Preasure (hPa)	NS	0.551	0.614	NS	0.187	0.235	0.307	0.331	0.316
Rainfall (mm)	− 0.374	NS	NS	− 0.374	− 0.368	− 0.365	NS	NS	NS
Sunsine duration (s)	0.253	NS	NS	0.252	0.382	0.210	0.462	0.402	0.351
Station	Zr								
Tmean (°C)	0.362	0.315	0.212	0.212	NS	NS	NS	NS	NS
Tmax (°C)	0.547	0.469	0.312	0.325	0.212	NS	NS	NS	NS
Tmin (°C)	0.521	0.382	0.278	NS	NS	− 0.220	− 0.420	− 0.449	− 0.348
Humidity (%)	NS	NS	NS	− 0.225	− 0.221	NS	NS	NS	NS
Wind speed (m/s)	NS	− 0.2445	− 0.291	NS	NS	NS	− 0.30215	NS	NS
Preasure (hPa)	0.358	0.375	0.377	NS	0.187	0.198	0.448	0.488	0.459
Rainfall (mm)	NS	− 0.253	− 0.214	− 0.323	− 0.344	− 0.287	NS	NS	NS
Sunsine duration (s)	0.260	0.245	NS	0.449	0.474	0.292	0.483	0.468	0.388

**Fig. 9:**
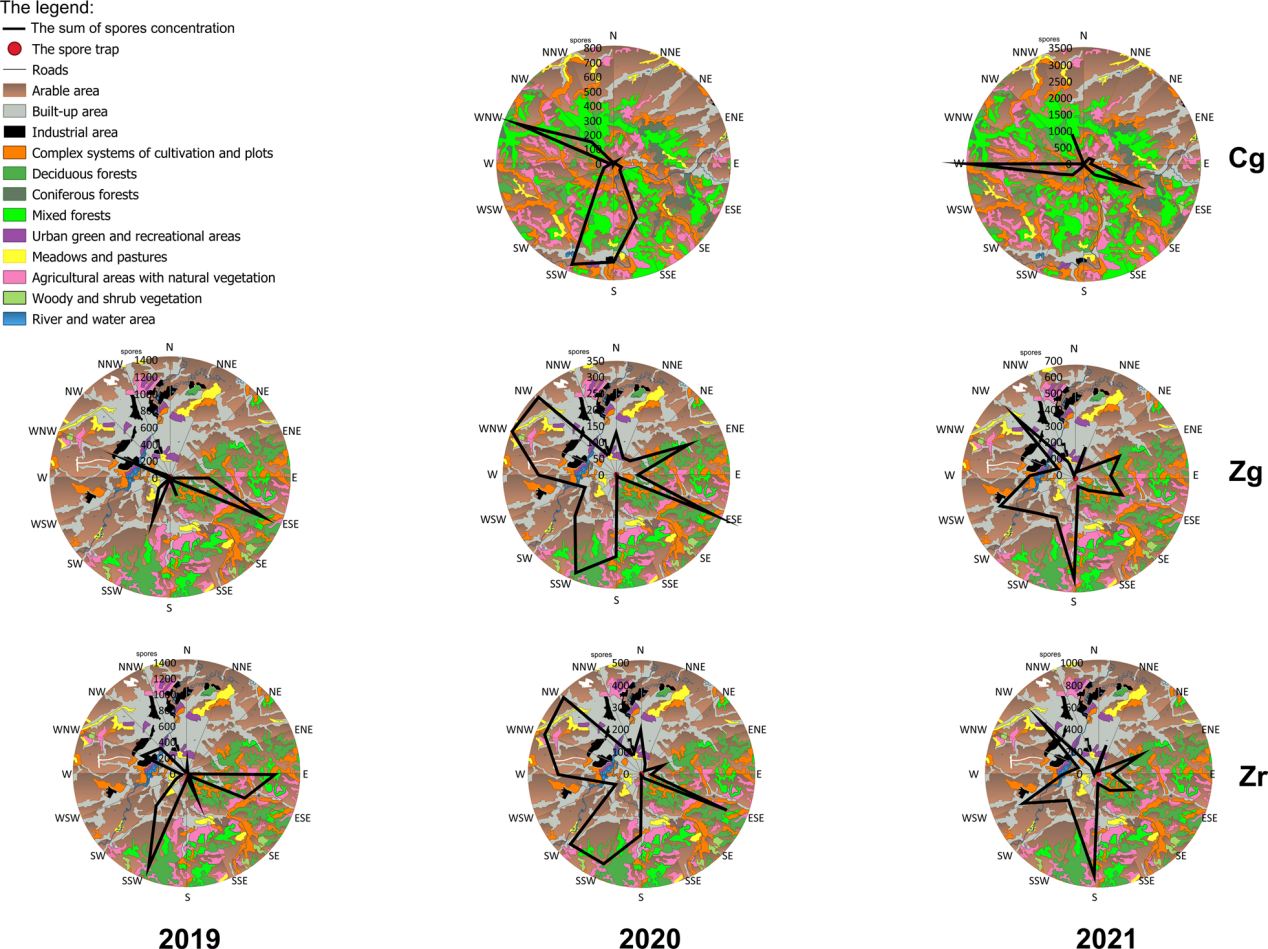
Sums of Boletus-type spores in days with a specific wind direction on the background of land cover types in three sites in 2019–2021

The fungal spore concentrations depended on the wind directions. At site Cg, the most spores were observed on days with the wind blowing from the south and from the northwest, from arable areas and agricultural areas with natural vegetation, all types of forests, and complex systems of cultivation and plots (Fig. 9; Additional file 3: Table S1). At the ground level station located in the city (Zg), in 2019 and 2020 high concentrations were noted on days with the south and east winds blowing from areas covered with deciduous and mixed forests, agricultural areas with natural vegetation, and complex systems of cultivation and plots. In 2021 spore concentration was dependent on the wind coming from the south (from arable areas, deciduous and mixed forests, agricultural areas with natural vegetation and built-up areas) and the northwest (from arable areas, built-up and industrial areas), and southwest (arable areas, complex systems of cultivation and plots, and built-up areas) directions (Fig. 8; Additional file 3: Table S1). At the other urban station located at roof level (Zr), in 2019 high spore concentrations, exceeding 1000 spores, were associated with the wind blowing from the east and the south directions, through arable areas and deciduous forests. In 2020 spore concentrations were linked to winds blowing over built-up areas, arable areas, deciduous forests, and complex systems of cultivation and plots. A year later, winds coming from the south (mainly from deciduous and mixed forests, agricultural areas with natural vegetation) contributed to the highest spore concentrations, greater than 900 spores (Fig. 9; Additional file 3: Table S1).

## DISCUSSION

### Temporal and spatial differences in the occurrence of Boletus-type spores in relation to the land cover

Boletes are frequently studied across the world, in particular in terms of their systematic affiliation and classification of species that are new to science (Pegler and Young 1981; Šutara and Skála 2007; Khmelnitsky et al. 2019). Studies on the aerobiology of their spores are scarce (Levetin 1990; Hernández Trejo et al. 2013; Grinn-Gofroń et al. 2020; Kilic et al. 2020). Levetin (1990, 1991) demonstrates that *Boletaceae* spores frequently occur in the air of Tulsa (central USA—a wet temperate climate). The high rainfall in May and June favors the appearance of fungi and their maximum spore concentrations predominantly occur in June. July and August are a dry period and spore concentrations of all basidiomycetes were low. In the Mediterranean subtropical climate zone (Spain, the cities of Mérida and Seville), Boletus-type spores are present in the air throughout the major part of the year and their high concentrations may appear even twice during the year—in early summer (June) and in autumn (November) (Hernández Trejo et al. 2013). The percentage of *Boletaceae* spores in the air spora is similar to that of *Phylacteria* (*Thelephoraceae*) or *Ganoderma* (*Ganodermataceae*) at more than 1%. In the continental climate of Turkey, airborne Boletus-type spores accounted for more than 5% of the fungal spores most frequently detected in the atmosphere. In early spring (March and April), these spores were not found in the air. An increase in spore concentrations was first recorded in May, but the highest concentrations of airborne spores were observed as late as the autumn—from September to November (Kilic et al. 2020). Grinn-Gofroń et al. (2020) expanded their study on the concentration of airborne Boletus-type spores to include the Black Sea region in Turkey, investigating their concentrations at several sites. They showed that the season start dates and the peak period of Boletus-type spore concentrations differed between the sites by even more than 130 days.

So far, the seasonality of occurrence of these spores in air in a warm temperate climate has not been described. Our study proves that in such climatic conditions the first Boletus-type spores appear in the air in May, whereas the period of their continuous occurrence begins in June, but their concentrations are not high. Peak concentrations are observed two or three times during the year, most frequently in late summer and in autumn when favorable conditions for the development of basidiomes of these mushrooms occur. This is a different scenario to that described by the above authors. In warmer climates, the increase of airborne spore concentrations occurs earlier and there is a physiological drought in the summer, which is not observed in temperate climates. A detailed analysis of the seasonality of these mushrooms’ spores in regions with different land use reveals relatively low variation in seasonal timing, but high variation in the intensity of spore release into the air between years. In 2019 the intensity of the season was higher than in the other two years; the seasonal spore integral (SSIn) at the sites ranged from 5 700 to nearly 14 000; in the next year, however, we recorded the lowest SSIn values. The annual total sum of Boletus-type spores recorded in Turkey was 7527 (Kilic et al. 2020). Distinctly lower concentrations were observed in Spain where—in Mérida they ranged from 710 to 3 485 (Hernández Trejo et al. 2013).

High concentrations of Boletus-type spores detected in the areas under study, especially during their seasonal peak, may be of significance for sensitive people. The literature of the subject reports cases of not only food allergies, but also contact and inhalant allergies (Helbling et al. 2002; Baruffini et al. 2005; Molin et al. 2015). The frequency of allergies to *Boletus* allergens is not high, but cases of cross reactions with the spores of, e.g., *Pleurotus* and *Coprinus* have been described (Lehrer et al. 1986; Helbling et al. 1998). Therefore, we consider that specific studies on the aerobiology of their spores and allergic responses are merited.

The presence of fungi is strictly related to many factors, including the habitat in which they grow. In boletes, mycelium germination can often be stimulated by tree root exudates, e.g. abietic acid released from the roots of pine trees (*Pinus sylvestris*) (Fries 1984; Fries et al. 1987). Fries (1978) also reports that basidiospore germination can be induced by volatile factors released by the mushrooms themselves and/or an addition of activated charcoal, but it was difficult to unambiguously determine which of these supplements distinctly affected spore germination. The strong host-tree dependence also influences the occurrence of basidiomes in these mushrooms and in consequence the discharge of spores and their concentration in the air. We proved that the spatial differences in Boletus-type spore concentrations between the stations located at ground level (Zg, Cg) particularly relates to their intensity. Higher spore concentrations were generally recorded in the countryside than in the city as would be predicted. Forests near the village in question, with a predominance of *Fagus* or *Quercus*, could have favored germination, and eventually basidiome formation of ectomycorrhizal *Boletaceae* (Wang et al. 1995, 1998) and so release of their spores into the air. Our study confirms that in this region as much as 27% of the area within the 10 km buffer promoted the occurrence of boletes. We also showed that at the site located in the countryside the spore concentration in 2020 increased when the wind was blowing from the directions where the area was covered with deciduous forests and complex systems of cultivation and plots. On the other hand, the number of spores was found to decrease when the wind was blowing from arable areas. Land cover dependent differences in spore concentration have already been documented in earlier studies. Kasprzyk and Worek (2006) demonstrated that the average concentrations of airborne fungal spores in the countryside were higher than in the city and that the type of land use, with high probability, could have affected the pattern of occurrence of spores or their daily average concentrations. A higher spore concentration was also recorded in a rural area than in an urban agglomeration in Portugal, in a warmer climate zone (Oliveira et al. 2009). Another study reveals that the richness and abundance of ectomycorrhizal fungi, also including *Boletaceae*, in oak woodland can be strongly dependent on agro-silvo-pastoral exploitation and tree mortality (Azul et al. 2010). Our study confirms he finding of Rojo et al. (2019) that the height at which aerobiological monitoring is carried out is an important factor influencing the results. At roof level, we always found a higher SSIn value and the airborne spore concentration in individual months was usually higher than at ground level. The differences between the concentrations of airborne spores at different heights may be due to several reasons. Forest is a natural habitat for the production of Boletus-type spoand while the number of urban parks is not large; only 13% of the city’s area had habitats that would be expected to favoring the occurrence of these fungi. Small spores floating in the air could have been carried by the wind blowing at higher heights from the suburban forests in the direction of the city. Khattab and Levetin (2008) demonstrated that the difference in spore concentrations at different heights may depend on the type of spore. They conducted a study in which they showed significantly higher concentrations of basidiospores, Penicillium/Aspergillus-type spores, and smut spores at ground level as well as higher concentrations of *Alternaria* and ascospores at the rooftop level. Furthermore, smaller spores may be predominant at greater heights, whereas larger spores may be more prevalent at lower levels (Chakraborty et al. 2001). An important factor is also the height at which the spores are released (Norros et al. 2014). Dąbrowska-Zapart and Niedźwiedź (2020), in turn, documented that the highest concentrations of fungal spores, monitored at different heights may also be influenced by weather factors, i.e. wind speed, maximum wind gust, and solar radiation. None of the authors cited here, however, examined the issue in relation to Boletus-type spores.

### Circadian periodicity of Boletus-type spores

Studies on the diurnal pattern of occurrence of basidiospores in the air indicate that most frequently their highest concentrations are found after midnight and during early morning hours when humidity conditions favour their release (Lyon et al. 1984; Calderon et al. 1995; Oliveira et al. 2009). Such a rhythm is characteristic of, for example, *Sporobolomyces*, *Agrocybe*, *Amanita*, *Coprinus*, and *Ganoderma* (Hirst 1953; Ščevková et al. 2019; Lagomarsino Oneto et al. 2020) as well as for representatives of boletes (Haard and Kramer 1970; Li 2005). We showed that the concentration of airborne Boletus-type spores increased twice within 24 h, the first increase in the early morning and the other at night. It should, however, be remembered that the diurnal cycle of occurrence of spores in the air is not always a faithful reflection of their release pattern (Ščevková et al. 2019). Other factors, e.g. the weather pattern, microclimate, land use, turbulent or convective air movements, have a great impact on the occurrence of airborne spores (Ingold 1971; Corden and Millington 2001; Grinn-Gofroń et al. 2018). Kasprzyk (2006) reports that in an agricultural area, where theoretically there is more plant material on which fungi may develop, the diurnal pattern of airborne conidia is closely related to the diurnal periodicity of sporulation and detected spores originate from very numerous local sources. Our study seems to confirm this thesis for boletes spores—the higher above ground level, the more the peak concentration hour is delayed.

### Effect of meteorological factors on the occurrence of airborne Boletus-type spores

Research regarding the effect of weather on the pattern of occurrence of airborne fungal spores has been frequently undertaken and researchers’ interest has predominantly focused on conidia (*Alternaria*, *Cladosporium*) and ascospores (*Leptosphaeria*), and amongst basidiomycetes, mainly basidiospores of *Ganoderma* (Hasnain 1993; Dawidziuk et al. 2012; Grinn-Gofroń et al. 2018). We agree that temperature and air humidity are the main parameters influencing airborne spores concentration. Our study complements sparse knowledge on the relationship between weather conditions and airborne spore concentrations in pileate mushrooms. We demonstrated that Boletus-type spore concentration in the air was correlated with the meteorological conditions and that temperature appeared to be the main weather factor that may initiate the growth of basidiomes and so spore release.

An increase in temperature could also have affected the dispersal of basidiospores since we found a positive correlation between thermal conditions and the concentration of bolete spores on the days preceding the day of measurement. Air humidity was also an important factor apparently influencing Boletus-type spore concentration. Humidity could affect the spore release mechanism and so the increase in real-time spore concentration. The importance of this parameter was underlined by Liu et al. (2017). Elevated humidity could favor the formation of Buller’s drop, which is an essential part of spore discharge into the environment (Webster et al. 1989). The study of Grinn-Gofroń et al. (2020) in a different climatic zone, the Black Sea region, similarly confirmed the importance of temperature and humidity for the phenomenon described.

The role of rainfall depends on its frequency, amount, and intensity (Fitt et al. 1989; Hjelmroos 1993). Kilic et al. (2020) did not show precipitation to have a statistically significant impact on the occurrence of airborne Boletus-type spores, and our study seems to only partially confirm this thesis. In the countryside, rainfall did not affect the occurrence of bolete spores, whereas in the city such a correlation was found, mainly in 2019. This divergence could relate to the different amounts of rainfall—in the city this was lower than in the rural area, and the correlation was negative. Rainfall may wash down spores floating in the air spora and so cause their deposition; this can be evidenced by the negative correlation found in our study.

Another parameter influencing airborne spore concentration could be wind speed. It is difficult to unambiguously determine the effect of wind on the concentration of palynomorphs. A strong wind may disperse them, but if the wind is persistently from a direction where sources of fungal mycelia are located, concentrations may rise. Our study confirms a decrease in Boletus-type spore concentrations with a strong wind, except where this is from the direction of forests,i.e. areas potentially favoring the occurrence of boletes, when their concentrations. It appears that wind could have been the factor responsible for the migration of spores between different habitat types. Our research demonstrates that the most favorable days for the occurrence of high Boletus-type spore concentrations were warm days without rainfall and with strong winds, but also with a relatively high air humidity.

## CONCLUSIONS

Multidimensional analysis is necessary to properly identify the aerobiology of basidiospores. In the case of boletes, we found that meteorological conditions (temperature, sunshine, humidity, and wind direction), as well as the type of land cover, were undoubtedly important factors determining the occurrence of Boletus-type spores in the air. Forests and areas of green vegetation are the main sources of airborne spores. In a warm temperate climate the seasonality of Boletus-type spores was similar to that of other basidiospores. The period of continuous occurrence occurs from June with the highest concentrations two or three times a year, most often in late summer and autumn, but each year the seasons may exhibit a different intensity. In the city, higher concentrations were recorded on the roof of the building than near the ground. Boletus-type spores also exhibit the periodicity of airborne spore behaviour characteristic for *Basidiomycota*, which at ground level corresponds to the sporulation rhythm—distinct periods of maximum concentrations (the first in the early morning and the second in the evening or at night) and their decrease. Due to their common incidence in the air, it could be worth including boletes spores along with other basidiospores in seasonal calendars for those with allergic reactions to pollen and/or spores. It is worth stressing that in our study area the concentration of Boletus-type spores can be so high as to merit mention in forecasts for allergy sufferers, especially as the period of their occurrence coincides with the time when boletes are collected by mushroom pickers. Multifaceted analysis of aerobiological data may support physicians in determining the etiology of inhalant allergies.

There remains, however, a very poor understanding of the *Boletus* aerobiology, and our research and analyses, although detailed and providing new insights into many aspects of this topic, require continuation and research on a larger spatial scale and in different regions.

### Supplementary Information

**Additional file 1. Fig. S1**: Mean temperature and rainfall in Rzeszów in 2019–2021.

**Additional file 2. Fig. S2**: Mean temperature and rainfall in Czudec in 2019–2021.

**Additional file 3. Table S1**: The type land cover [ha] of studied areas in Czudec village and Zalesie (Rzeszów city) in the 10 km buffers divided into 16 sectors designated in the relation of world directions. 

## Data Availability

The data are available from the corresponding authors upon request.

## Abbreviations

st.dt: Start of the season
st.jd: Day of the year of start season
en.dt: End of the season
en.jd: Day of the year of end season
fr: Frequency (%)
ln.s: Length of the season
sm.tt: Total sum
SSIn: Sesonal spores integral
pk.val: Peak value
pk.dt: Data of the peak value
pk.jd: Day of the year of peak value
daysth: Number of days with more than 100 spores
