# Supplementary figures and images for: Seasonality and intensity of airborne Boletus-type spores in relation to land use and weather pattern
Source: IMA Fungus. 2023 Dec 20;14:26. doi: 10.1186/s43008-023-00135-4 (PMC10734109; doi:10.1186/s43008-023-00135-4)

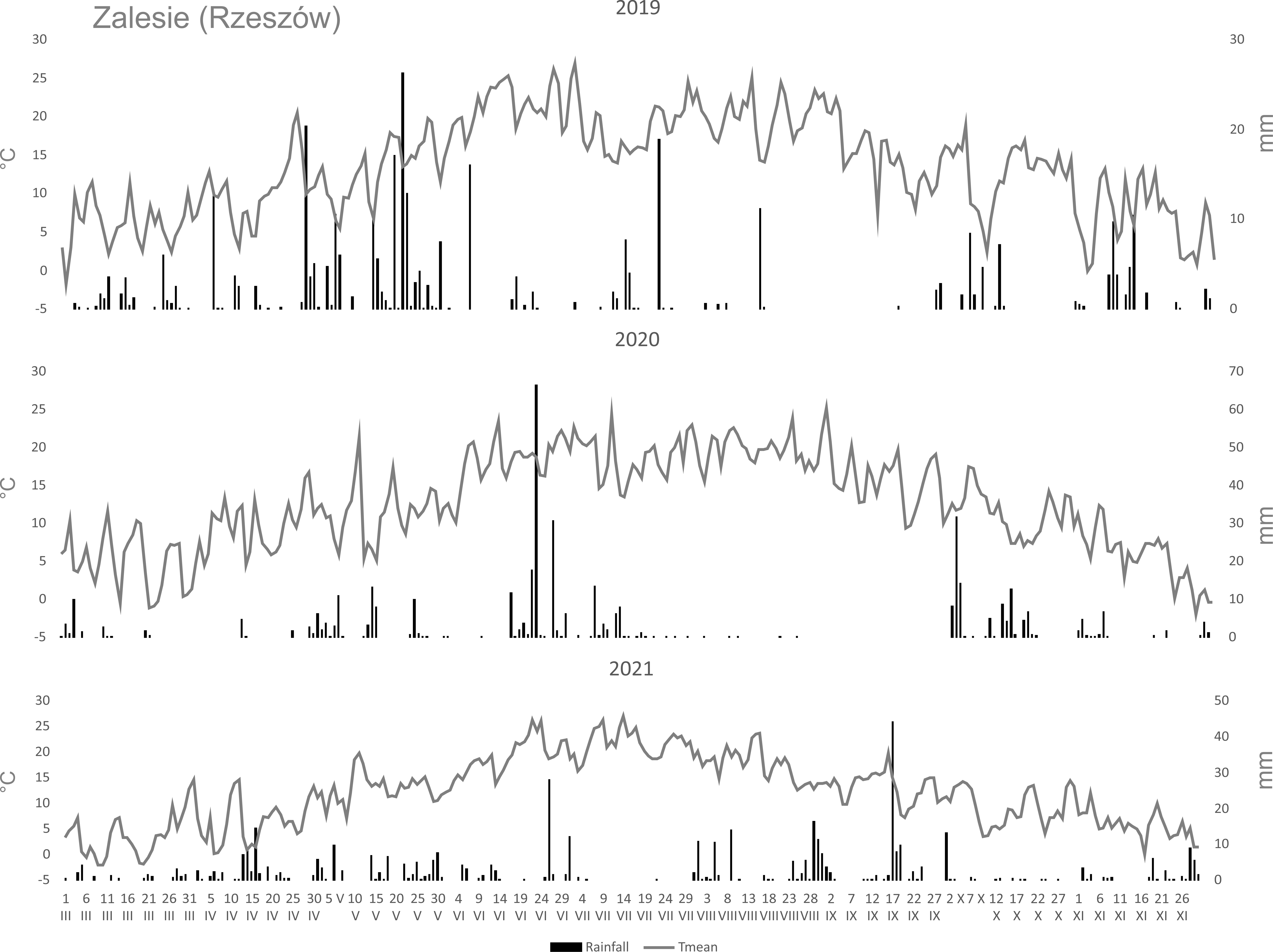

Supplement: Supplementary file 1 — Additional file 1. Fig. S1: Mean temperature and rainfall in Rzeszów in 2019–2021. [file 43008_2023_135_MOESM1_ESM.jpg]

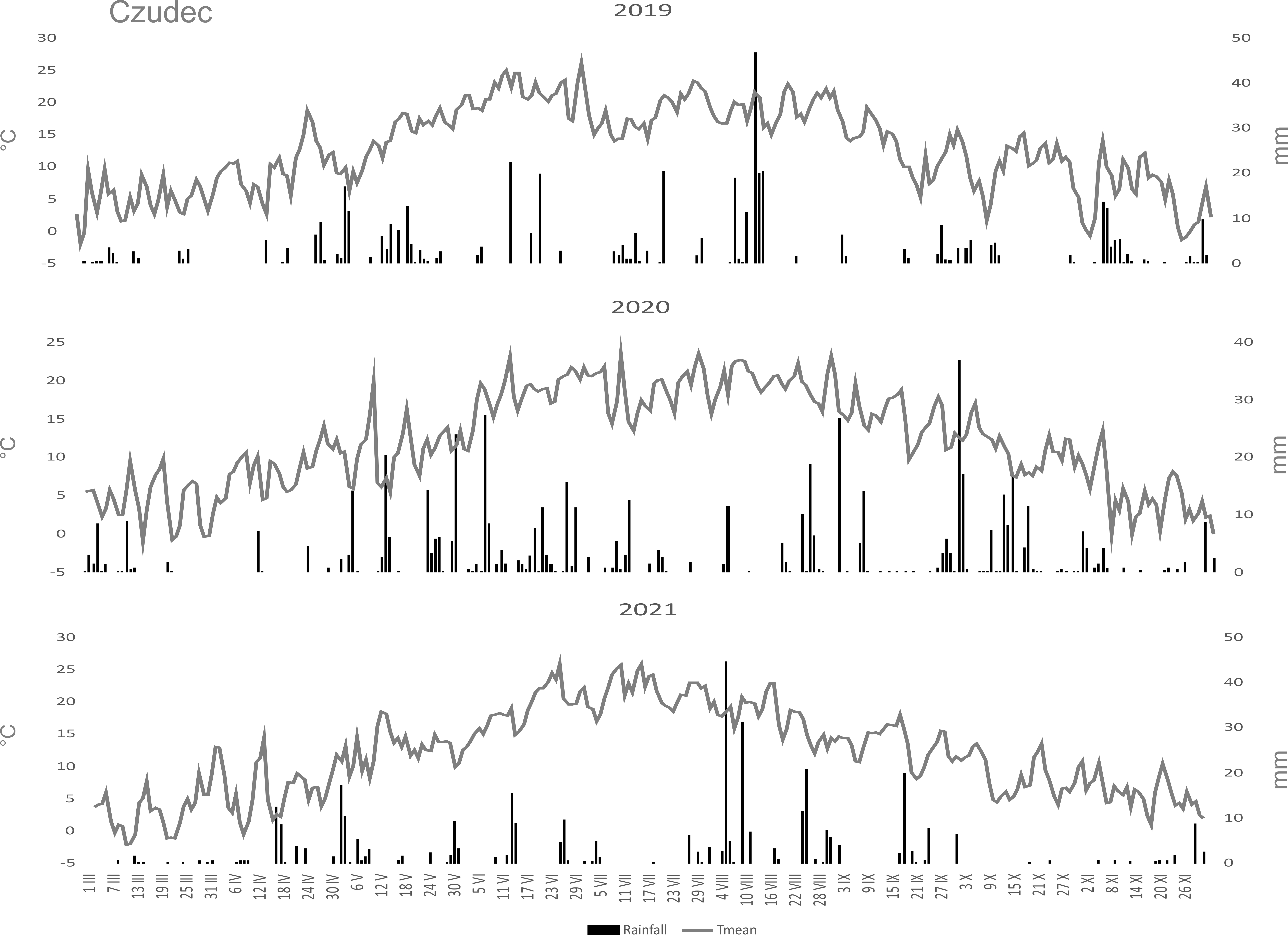

Supplement: Supplementary file 2 — Additional file 2. Fig. S2: Mean temperature and rainfall in Czudec in 2019–2021. [file 43008_2023_135_MOESM2_ESM.jpg]
