# Supplementary material for: Seasonality and intensity of airborne Boletus-type spores in relation to land use and weather pattern
Source: IMA Fungus. 2023 Dec 20;14:26. doi: 10.1186/s43008-023-00135-4 (PMC10734109; doi:10.1186/s43008-023-00135-4)
Supplement: Supplementary file 3 — Additional file 3. Table S1: The type land cover [ha] of studied areas in Czudec village and Zalesie (Rzeszów city) in the 10 km buffers divided into 16 sectors designated in the relation of world directions. [file 43008_2023_135_MOESM3_ESM.docx]

**Table 1S.** The type land cover [ha] of studied areas in Czudec village and Zalesie (Rzeszów city) in the 10 km buffers divided into 16 sectors designated in the relation of world directions; types of land use marked in red were assumed as favorable for the occurrence of boletes spores

| **The sector** | | **N** | **NNE** | **NE** | **ENE** | **E** | **ESE** | **SE** | **SSE** | **S** | **SSW** | **SW** | **WSW** | **W** | **WNW** | **NW** | **NNW** |
| --- | --- | --- | --- | --- | --- | --- | --- | --- | --- | --- | --- | --- | --- | --- | --- | --- | --- |
| **The type of land use [Ha]** |  | **Czudec** | | | | | | | | | | | | | | | |
|  | Arable area | 1237.1 | 1174.1 | 1012.5 | 922.0 | 715.8 | 557.2 | 759.0 | 463.8 | 512.1 | 803.2 | 801.4 | 970.3 | 739.4 | 777.9 | 847.2 | 1078.6 |
|  | Meadows and pastures | 66.6 | 63.6 |  | 75.5 | 16.2 | 31.6 | 25.5 | 54.5 | 20.2 | 83.0 | 25.4 | 74.4 |  |  | 98.7 | 105.2 |
|  | Coniferous forests |  | 70.0 | 122.2 | 117.1 | 101.2 | 292.2 | 69.0 |  |  |  |  |  | 86.1 | 3.9 |  |  |
|  | Deciduous forests | 59.3 | 25.7 | 54.4 | 1.6 | 25.8 | 26.9 |  | 82.8 | 124.4 | 35.0 | 263.9 | 300.9 | 106.2 | 288.1 | 111.3 | 64.1 |
|  | Mixed forests | 235.3 | 48.1 | 242.7 | 321.7 | 333.5 | 550.0 | 636.8 | 454.2 | 556.8 | 326.2 | 201.0 | 68.9 | 522.7 | 577.8 | 555.8 | 311.8 |
|  | Urban green and recreation area |  |  |  |  |  |  |  |  | 48.8 |  |  |  |  |  |  |  |
|  | Water area |  |  |  |  |  |  |  |  |  | 26.5 |  |  |  |  |  |  |
|  | Industrial area |  |  | 8.9 |  |  |  |  | 9.0 | 30.3 |  |  |  |  |  |  |  |
|  | Agricultural areas with natural vegetation | 168.8 | 147.9 | 70.2 | 66.5 | 115.8 | 99.2 | 199.8 | 409.3 | 112.3 | 307.7 | 296.7 | 272.7 | 307.8 | 143.8 | 51.5 | 90.0 |
|  | Built-up area | 170.2 | 307.9 | 324.9 | 342.9 | 331.7 |  | 36.7 | 97.5 | 270.5 | 183.8 |  |  |  |  | 82.0 | 185.1 |
|  | Woody and shrub vegetation |  |  |  |  | 32.1 | 24.6 | 35.6 |  |  |  |  |  |  |  |  |  |
|  | Complex systems of cultivation and plots | 27.6 | 126.4 | 124.4 | 115.4 | 288.9 | 383.0 | 199.8 | 392.4 | 287.2 | 198.2 | 376.6 | 273.9 | 201.4 | 168.1 | 218.8 | 128.9 |
|  |  | Zalesie (Rzeszów) | | | | | | | | | | | | | | | |
|  | Arable area | 754.13 | 1012.7 | 1114.0 | 743.0 | 746.3 | 453.8 | 768.7 | 491.2 | 547.1 | 1144.1 | 1275.5 | 1041.7 | 979.6 | 674.4 | 455.7 | 438.86 |
|  | Meadows and pastures | 136.29 | 121.9 | 25.7 |  |  | 0.9 | 96.9 | 68.5 |  | 31.3 | 89.0 | 28.5 | 41.0 | 185.5 | 37.3 | 55.223 |
|  | Coniferous forests |  |  |  |  |  |  |  |  |  |  |  |  |  |  |  |  |
|  | Deciduous forests | 62.338 |  | 183.6 | 641.4 | 180.9 | 752.0 | 248.3 | 252.0 | 582.3 | 132.3 |  |  |  |  |  |  |
|  | Mixed forests |  |  | 101.6 | 117.5 | 203.6 | 56.8 | 173.6 | 398.0 | 136.8 | 105.1 |  |  |  |  |  |  |
|  | Urban green and recreation area | 106.16 | 5.9 |  |  |  |  |  |  |  |  |  |  | 33.4 | 46.8 | 98.2 | 149.46 |
|  | Water area |  |  |  |  |  |  |  |  |  |  |  | 42.5 | 55.4 | 24.2 | 2.8 |  |
|  | Industrial area | 85.555 |  |  |  |  |  |  |  |  |  |  | 53.1 | 103.4 | 142.0 | 151.6 | 282.74 |
|  | Agricultural areas with natural vegetation |  |  |  | 65.9 | 352.7 | 179.0 | 194.4 | 455.0 | 424.3 | 158.8 | 12.8 |  | 65.0 | 46.1 | 28.3 | 153.42 |
|  | Built-up area | 799.63 | 722.9 | 445.4 | 105.9 | 209.9 | 21.7 | 97.3 | 214.8 | 216.7 | 343.6 | 546.8 | 560.4 | 437.1 | 769.4 | 1121.5 | 833.83 |
|  | Woody and shrub vegetation |  |  |  |  |  | 61.7 | 86.3 |  |  | 44.0 |  |  | 19.9 | 8.6 |  |  |
|  | Complex systems of cultivation and plots | 12.447 | 96.7 | 93.3 | 289.1 | 269.2 | 436.4 | 297.9 | 82.2 | 53.8 | 5.4 | 38.9 | 236.4 | 174.9 | 64.4 | 0.4 | 56.921 |
